# Genetic spectrum of retinal dystrophies in Tunisia

**DOI:** 10.1038/s41598-020-67792-y

**Published:** 2020-07-08

**Authors:** Imen Habibi, Yosra Falfoul, Ahmed Turki, Asma Hassairi, Khaled El Matri, Ahmed Chebil, Daniel F. Schorderet, Leila El Matri

**Affiliations:** 1 IRO-Institute for Research in Ophthalmology, Av du Grand-Champsec 64, 1950 Sion, Switzerland; 2 Oculogenetic Laboratory LR14SP01, Hedi Rais Institute of Ophthalmology (Department B), Tunis, Tunisia; 3 Faculty of Biology and Medicine, University of Lausanne, Lausanne, Switzerland; 4 Faculty of Life Sciences, Ecole Polytechnique fédérale de Lausanne, Lausanne, Switzerland

**Keywords:** Genetic testing, DNA damage and repair

## Abstract

We report the molecular basis of the largest Tunisian cohort with inherited retinal dystrophies (IRD) reported to date, identify disease-causing pathogenic variants and describe genotype–phenotype correlations. A subset of 26 families from a cohort of 73 families with clinical diagnosis of autosomal recessive IRD (AR-IRD) excluding Usher syndrome was analyzed by whole exome sequencing and autozygosity mapping. Causative pathogenic variants were identified in 50 families (68.4%), 42% of which were novel. The most prevalent pathogenic variants were observed in *ABCA4* (14%) and *RPE65*, *CRB1* and *CERKL* (8% each). 26 variants (8 novel and 18 known) in 19 genes were identified in 26 families (14 missense substitutions, 5 deletions, 4 nonsense pathogenic variants and 3 splice site variants), with further allelic heterogeneity arising from different pathogenic variants in the same gene. The most common phenotype in our cohort is retinitis pigmentosa (23%) and cone rod dystrophy (23%) followed by Leber congenital amaurosis (19.2%). We report the association of new disease phenotypes. This research was carried out in Tunisian patients with IRD in order to delineate the genetic population architecture.

## Introduction

Inherited retinal dystrophies (IRD) are a large group of inherited eye disorders which affect photoreceptors and lead to visual impairment. The prevalence of IRD has been estimated in one case for each 2,500–7,000 persons among the general population^1^. IRDs are further classified into as retinitis pigmentosa (RP), cone rod dystrophy (CRD), and cone dystrophy (CD). Initial symptoms include night blindness, photophobia and/or progressive loss of the peripheral vision^2^. Clinical symptoms vary across different IRD subtypes and different disease genes.

Genetically, different IRD can be caused by pathogenic variants in more than 300 genes, over 100 of these have been linked to syndromic IRD (https://sph.uth.edu/retnet/), displaying three form of inheritance: autosomal dominant (AD), autosomal recessive (AR) and X-linked (XL). Occasionally, mitochondrial variants and digenic inheritance have been identified^3^.

Molecular genetics is essential for gene-based treatment, clarify diagnoses and to direct appropriate counseling. However, it is currently unknown how many genes are involved in IRDs, and even by using the latest next generation sequencing (NGS) techniques, pathogenic and likely pathogenic variants are identified only in 50% to 75% of patients^4^. Due to the relatively high frequency of consanguinity in Tunisia, ranging from 20 to 40%, this population could contribute to the identification of new genes responsible for AR-IRD^5^. To identify causative pathogenic variants in a large cohort of families diagnosed with nonsyndromic (24/26) or syndromic (2/26) AR-IRD, homozygosity mapping of known IRD loci was carried out. Pathogenic variant screening of the identified genes in all 74 families gave an overall idea about the most frequent genes and variants in patients with IRD in Tunisia. We believe it is essential to combine molecular and clinical data to diagnose IRD patients, especially with the emergence of therapeutic options.

## Results

### Clinical diagnosis and pathogenic and likely pathogenic (P/LP) variants identified

50 affected and 48 unaffected relatives belonging to 26 families with suspected recessive inheritance were included. Pathogenic variants are listed in Table 1. A total of 26 causative P/LP variants in 19 genes were identified in 26 families, including 14 missense substitutions (53.9%), 5 deletions (19.2%), 4 nonsense P/LP variants (15.4%) and 3 splice site pathogenic variants (11.5%). 8 (30.8%) P/LP variants were novel, while the remaining 18 (69.2%) were reported previously. 96.2% of all P/LP variants were homozygous, only one family carried a heterozygous pathogenic variant in *PRPH2* in family 17 (F17) (3.8%). Segregation of the mutant allele was confirmed in the majority of the families. For missense variant the substituted amino acid residues are highly conserved across species, and in silico pathogenicity prediction tools PolyPhen2 and SIFT predicted these changes to be deleterious.

**Table 1:** Pathogenic variants identified in this study.

Family ID	Disease	Genotyping Method	Size of homozygous region, in Mb	Chr	Gene	DNA pathogenic variant	Predicted protein variant	Reference sequence	Previously reported	SIFT	PolyPhen
F1	LCA	WES	–	14q11.2	***RPGRIP1***	c.[3113-3114delCT];[3113-3114delCT]	p.[T1038Rfs*8]; T1038Rfs*8]	NM_020366	This study	–	–
F2	LCA	IROme	–	17p31.1	***GUCY2D***	c.[2660 T > G];[2660 T > G]	p.[V887G];[V887G]	NM_000180	This study and^6^	0	0.999
F3	LCA	Asper	–	1p31.3	*RPE65*	c.[700C > T];[700C > T]	p.[R234*];[R234*]	NM_000329	^29^	–	–
F4	LCA	WES	–	3q13.33	*IQCB1*	c.[994C > T];[994C > T]	p.[R332*];[R332*]	NM_001023570	^30^	–	–
F5	LCA	WES	–	1q31.3	***CRB1***	c.[3542 + 1G > A];[3542 + 1G > A]	–	NM_201253.2	This study	–	–
F6	CRD	WES	40	1q31.3	*CRB1*	c.[2506C > A];[2506C > A]	p.[P836T];[P836T]	NM_201253.2	^31^	0.04	0.999
F7	CRD	WES	124	1q31.3	*CRB1*	c.[ 2105A > G];[ 2105A > G]	p.[Y702C];[Y702C]	NM_201253.2	^32^	0	0.89
F8	CRD	WES	–	10q23.1	***CDHR1***	c.[863-2_863-1delAG];[863-2_863-1delAG]	–	NM_033100	This study	–	–
F9	CRD	WES	–	8q22.1	***C8ORF37***	c.[470 + 1G > T];[470 + 1G > T]	–	NM_177965	This study	–	–
F10	CRD	WES	–	2p23.2	*C2ORF71*	c.[2756_2768del13];[ 2756_2768del13]	p.[K919Tfs*2];[ K919Tfs*2]	NM_001029883	^33^	–	–
F11	CRD	WES	35	1p22.1	***ABCA4***	c.[1916A > G];[1916A > G]	p.[Y639C];[Y639C]	NM_000350.2	This study	0.01	1
F12	RP	WES	77	1p22.1	*ABCA4*	c.[4139C > T];[4139C > T]	p.[P1380L];[P1380L]	NM_000350.2	^34^	0	0.716
F13	STGD	WES	–	1p22.1	*ABCA4*	c.[1140 T > A];[1140 T > A]	p.[N380K];[N380K]	NM_000350.2	^35^	0.01	0.05
F14	STGD	WES	–	1p22.1	*ABCA4*	c.[3259G > A];[3259G > A]	p.[E1087K]; [E1087K]	NM_000350.2	^36^	0	0.999
F15	CRD/STGD	WES	–	1p22.1	*ABCA4*	c.[3259G > A];[3259G > A]	p.[E1087K]; [E1087K]	NM_000350.2	^36^	0	0.999
F16	RP	WES	–	1p36.22	*NMNAT1*	c.[37G > A];[37G > A]	p.[A13T];[A13T]	NM_001297778.1	^8^	0	1
F17	RP	WES	–	6p21.1	*PRPH2*	c.[133C > T];[ =]	p.[L45F];[ =]	NM_000322	^37^	0	0.991
F18	RP	WES	–	2p15	*FAM161A*	c.[685C > T];[685C > T]	p.[R229*];[R229*]	NM_001201543	^38^	–	–
F19	RP	WES	–	16q21	***CNGB1***	c.[2293C > T];[2293C > T]	p.[R765C];[R765C]	NM_001297	This study and ^6^	0	0.999
F20	RP	WES	–	6q12	*EYS*	c.(1766 + 1_1767-1)_(2023 + 1_2024-1)del	–	NM_001292009	^39^	–	–
F21	RP	WES	–	6q12	*EYS*	c.[5928-2A > G];[5928-2A > G]	–	NM_001292009	^9^	–	–
F22	SBB	WES	–	2q31.1	*BBS5*	c.[214G > A];[214G > A]	p.[G72S];[G72S]	NM_152384.2	^40^	0	1
F23	SBB	WES	48	2q31.1	*BBS5*	c.[123delA];[123delA]	p.[G42Efs*11];[ G42Efs*11]	NM_152384.2	^41^	–	–
F24	ACHM	WES	119	2q11.2	*CNGA3*	c.[1114C > T];[1114C > T]	p.[P372S];[P372S]	NM_001298.2	^42^	0	0.989
F25	ACHM	WES	87c	8q21.3	*CNGB3*	c.[1810C > T];[1810C > T]	p.[R604*];[R604*]	NM_019098.4	^43^	–	–
F26	CSNB	WES	–	15q13.3	***TRPM1***	c.[3947 T > G];[3947 T > G]	p.[L1316R];[L1316R]	NM_002420.5	This study	0	0.075

Genes highlighted in bold harbor the novel pathogenic variants identified in this study.

LCA = Leber congenital amaurosis; RP = retinitis pigmentosa; CRD = cone-rod dystrophy; STGD = Stargardt disease; BBS = Bardet–Biedl syndrome; ACHM = Achromatopsia; CSNB = congenital stationary night blindness.

After molecular testing, all patients were re-evaluated to monitor whether their retinal phenotype was similar to previously described retinopathies caused by pathogenic variants in the same gene. In case of discrepancy, the respective phenotypes were considered as potential novel genotype–phenotype correlations.

Information for each patient is presented in Table 2. Below we present the families with novel P/LP variants.

**Table 2:** Summary of the clinical data of 26 families with gene-associated retinal dystrophies.

Family	Patient	Gender	Age years	Age of onset^a^	Visual acuity OD OS	Ophthalmoscopy	Optical coherence tomography	Full-Field ERG (ODS)	Diagnosis	Gene
F1	IV.7	M	30	Birth	LP LP	Vessel attenuation RPE mottling and spicule deposits from the mid-retina to the periphery Macula seems preserved		Extinct response	LCA	*RPGRIP1*
F2	II.2	M	4	Birth	LP LP	Normal fundus appearance		Extinct response	LCA	*GUCY2D*
F3	III.1	F	39	Birth	LP LP	Vessel attenuation RPE mottling and spicule deposits from the mid-retina to the periphery			LCA	*RPE65*
F4	III.1	F	8	Birth	1/20 RE/LE	Normal fundus appearance	Normal	Extinct response	LCA	*IQCB1*
	III.2	M	1	Birth	NM	Normal fundus appearance				
F5	II.1	F	8	Birth	LP + RE LP—LE	RE: preserved para-arteriolar RPE, Peripheral nummular pigment clumping and atrophy LE: Coats-like exudative Vasculopathy		Extinct response	LCA	*CRB1*
F6	II.1	M	48	10	HM	Cone-rod dystrophy with yellowich macular deposits Mid-peripheral nummular pigment clumping and atrophy	Macular atrophy		CRD	*CRB1*
F7	II.1	M	14	6	1/20	Cone-rod dystrophy with yellowich macular deposits nummular pigment clumping and atrophy	Macular disorganization and cysts		CRD	*CRB1*
F8	III.1	F	32	12	LP LP	Few bone spicule shaped deposits in the mid periphery along with atrophy of the periphery retina, Early macular atrophy	RE: macular hole LE: macular atrophy	Altered photopic and scotopic responses	CRD	*CDHR1*
	III.3	F	44	10	LP LP	Vessel attenuation RPE mottling and spicule deposits from the mid-retina to the periphery macular atrophy with spicule deposits	Macular atrophy			
F9	IV.4	M	30	10	1/10 1/20	Beaten-bronze aspect of the macula Peripheral RPE atrophy Mild optic atrophy, Narrowing of the Vessels	Macular atrophy	Altered photopic and scotopic responses	CRD	*C8ORF37*
	IV.6	F	32	8	HM HM		Macular atrophy			
	IV.2	M	52	Infancy	LP LP	Gliosis of the posterior pole Diffuse retinal atrophy	Macular atrophy with parafoveolar gliosis			
F10	II.1	F	43	18	1/10 RE 2/10 LE	Symmetrical cloverleaf maculopathy with patchy circular midperipheral RPE atrophy and nummular pigment deposits	Macular atrophy	Altered cone and rods ERG predominating on photopic responses	CRD	*C2ORF71*
	II.2	M	48	15	3/10 RE/LE					
	II.3	M	62	14	LP RE/LE					
F11	II.2	F	43	9	Finger count	Diffuse macular, peripapillary and RPE atrophy extending beyond the vascular arcades Hyperplasia of the RPE	Macular atrophy		CRD	*ABCA4*
F12	II.1	F	58	10	HM	Diffuse macular, peripapillary and peripheral RPE atrophy;	Macular atrophy	Altered ERG responses predominating on photopic waves	STGD	*ABCA4*
F13	II.2	F	14	6	1/10 RE/LE	Bull’s eye maculopathy yellowish deposits	Macular atrophy		STGD	*ABCA4*
F14	III.3	F	18	6	1/10 RE/LE	Bull’s eye maculopathy yellowish deposits	Macular atrophy		STGD	*ABCA4*
F15	II.1	F	19	Before five	HM	Bull’s eye maculopathy Peripheral RPE Atrophy and yellowish deposits	Macular atrophy	Altered photopic responses with slightly altered scotopic responses	STGD	*ABCA4*
	II.2	M	14	Before Five	Hand movement	Bull’s eye maculopathy Peripheral RPE Atrophy and yellowish deposits	Macular atrophy	Altered photopic responses with slightly altered scotopic responses		
F16	V.4	M	21	5	7/10 6/10	Few bone spicule shaped pigment deposits and white dot deposits in the mid periphery Narrowing of the vessels. Waxy optic discs	Normal		RP	*NMNAT1*
	V.1	F	23	5	5/10 5/10	Few bone Spicule shaped Pigment deposits and white dot deposits in the mid periphery Hyperplasia of the RPE				
F17	V.1	F	29	20	10/10 3/10	Typical RP changes with bone spicule shaped pigment deposits in the mid periphery along with normal retinal areas	Normal macula		RP	*PRPH2*
F18	IV.1	M	33	11	1/20 RE/LE	Rare bone Spicule shaped Pigment deposits Large areas of retinal atrophy around vessels	Macular atrophy		RP	*FAM161A*
	IV.3	F	28	18	10/10 RE/LE	Rare bone Spicule shaped Pigment deposits in mid periphery	Normal macula			
F19	IV.2	F	63	16	3/10 RE 1/10 LE	Typical RP changes with bone spicule shaped pigment deposits in the mid periphery	Normal macula		RP	*CNGB1*
F20	II.5	F	52	10	2/10 RE/LE	Typical RP changes with bone spicule shaped pigment deposits in the mid periphery Yellowish macular deposits	Atrophy		RP	*EYS*
F21	II.5	F	32	16	5/10 RE/LE	Typical RP changes with bone spicule shaped pigment deposits in the mid periphery	Normal		RP	*EYS*
F22	II.2	M	45	9	HM RE/LE	Cone-rod dystrophy with bone spicule deposits and atrophy in the posterior pole and peripheral retina	Macular atrophy		BBS	*BBS5*
F23	II.1	M	41	8	1/20	Rare bone spicule shaped pigment deposits in the mid periphery macular atrophy	Atrophy		BBS	*BBS5*
F24	II.3	M	36	Birth	1/10 RE/LE	Normal fundus examination High myopia	Normal macula		ACHM	*CNGA3*
F25	II.1	M	18	Birth	2/10 RE/LE	Normal fundus examination	Retrofoveolar ellipsoid dysruption		ACHM	*CNGB3*
F26	II.1	F	50	Before 5	2/10 RE /LE	High myopia, cataract Chorioretinal atrophy	Atrophy		CSNB	*TRPM1*

CF = counting fingers; HM = hand movements; LP = light perception; HM: hand movement.

RE = right eye; LE = Left eye; RLE = both eyes.

CRD = cone rod dystrophy; STGD = Stargardt macular degeneration; LCA = Leber congenital amaurosis; RP = retinitis pigmentosa; CSNB = congenital stationary night blindness; ACHM = Achromatopsia; BBS = Bardet–Biedl syndrome.

F = female; M = male; PP = posterior pole; RPE = retinal pigment epithelium.

#### LCA (Fig. 1, 2)

**Figure 1:**
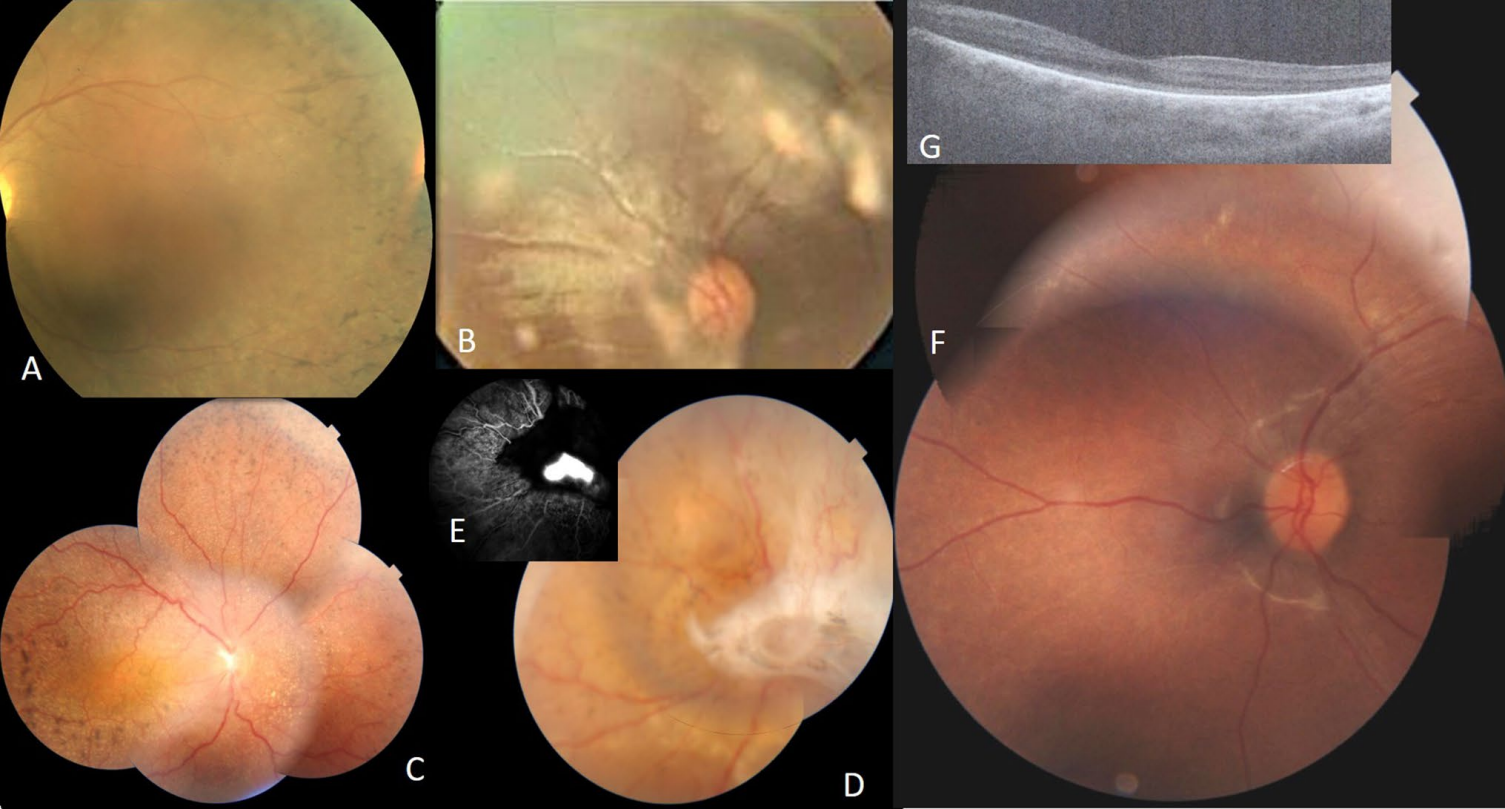
Clinical features of LCA patients; A F1 LE fundus of patient IV.7. B F2 RE fundus of patient II.2. C , D F5 fundus of RLE of patient II.1. E F5 fluorescein angiography of LE of patient III.1 showing noevascular membrane. F F4 LE fundus of patient III.1. G F4 OCT of LE of patient III.1.

**Figure 2:**
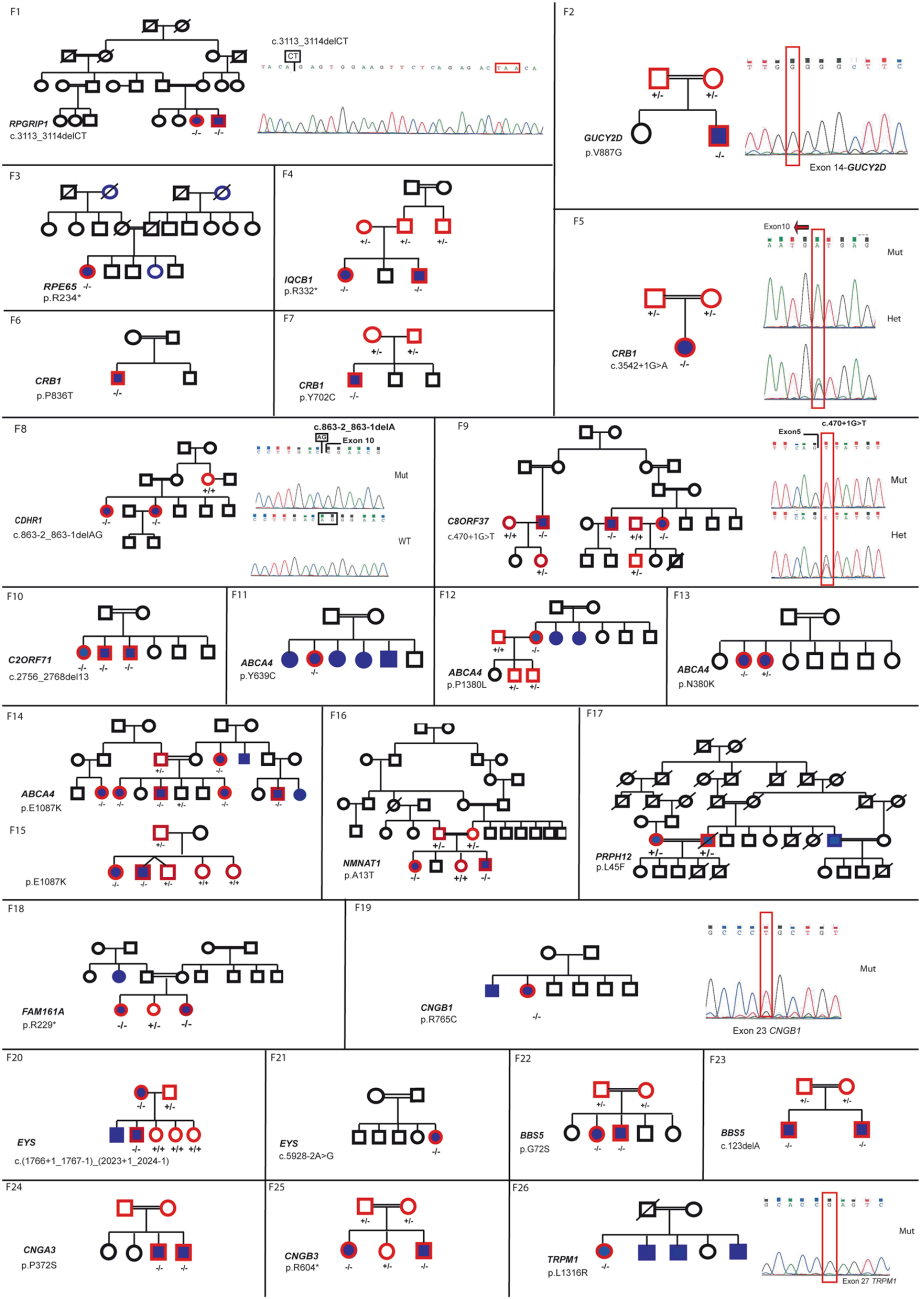
Segregation analysis of disease causing variants in the families with IRD. Affected individuals are indicated with filled symbols (blue), whereas unaffected relatives are indicated by open symbols. +: wild type allele; −: pathogenic variant.

Clinical data of patients from families F1, F2, F3, F4 and F5 revealed an age of onset of disease from birth with nystagmus and photophobia. BCVA was limited to light perception. Patient in F3 was monophthalmic of the right eye (RE) (Fig. 1).

#### F1-*RPGRIP1* (Fig. 1A)

One novel homozygous deletion (NM_020366: c.3113-3114delCT, p.T1038Rfs*8) in *RPGRIP1* was identified in F1 with 2 affected members. The deletion of the CT in exon 10 results in a frameshift with a premature stop codon at position amino acid 1046.

##### F2-*GUCY2D* (Fig. 1B)

The second novel homozygous likely pathogenic variant (NM_000180: c.2660 T > G, p.V887G) in *GUCY2D* was found in F2 in one affected individual. This likely pathogenic variant has previously been published in the validation of a targeted array but no phenotype was presented^6^.

##### F5-*CRB1* (Fig. 1C, 1D, 1E)

The novel homozygous likely pathogenic variant (NM_201253.2: c.3542 + 1G > A, p.?) in *CRB1* was found in F5 in one patient (Fig. 2). Fundus appearance in this proband included all clinical characteristics of *CRB1* pathogenic variant.

##### F4*-IQCB1*

The homozygous likely pathogenic variant (NM_001023570: c.994C > T, p.R332*) in *IQCB1* was found in F4 in two affected children (Fig. 1F and G). Fundus appearance in both probands revealed normal structure. Renal function and ultrasound were normal.

#### CRD patients (Fig. 2,3)

**Figure 3:**
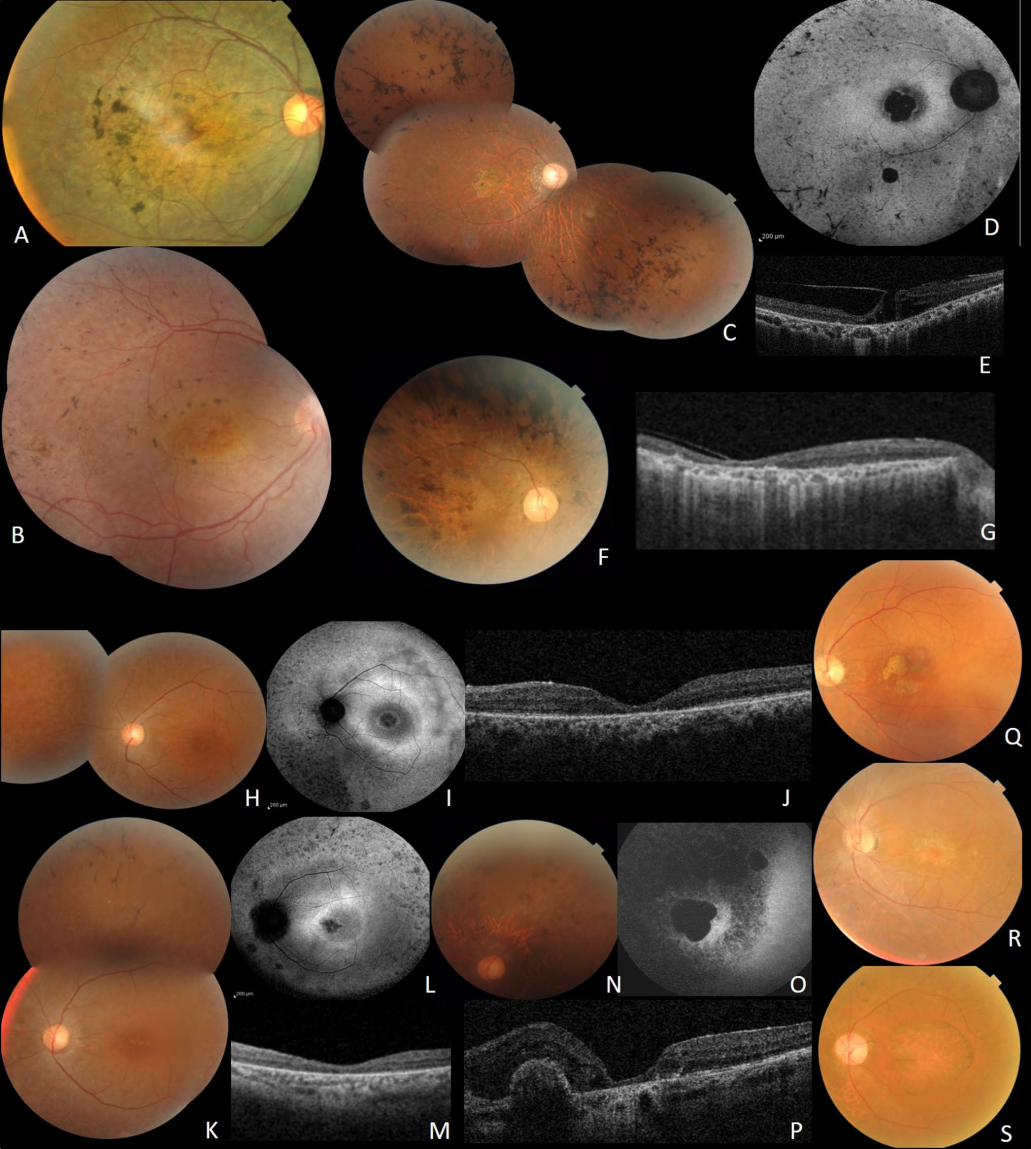
Clinical features of CRD patients; A F6, RE fundus of patient II.1. B F7, RE of patient II.1. C, D, E, F and G Clinical features of patients from F8. C: Fundus imaging of RE of index patient III.2. D FAF showing central macular hypoFAF surrounded by ring of hyper FAF, small areas of hypoFAF in the mid-periphery. E SS-OCT showing vitreo-retinal traction with macular hole. F fundus photo of the right eye of patient III.3. G SS-OCT showing diffuse chorio-retinal atrophy. H, I, J, K, L, M, N, O and P Clinical features of patients from F9. H Fundus imaging of LE of patient IV.4. I FAF showing central macular hypoFAF surrounded by ring of hyper FAF. J SS-OCT showing macular atrophy. K fundus photo of the left eye of the sister IV.6. L FAF showing central macular hypoFAF surrounded by ring of hyper FAF. M SS-OCT showing macular atrophy. N fundus photo of the left eye of IV.2. O FAF large macular atrophy surrounded smaller areas of atrophy. P SS-OCT showing macular atrophy with parafoveolar gliosis. Q, R and S Clinical features of patients from F9 showing cloverleaf maculopathy with peripheral RPE atrophy.

6 families (15 patients) with CRD (F6, F7, F8, F9, F10 and F11) were included. Their mean age was 35 years (14–48 years) with disease onset ranging from 6 to 18 years. All patients had photophobia, visual loss and night blindness. BCVA ranged from light perception to 2/10. Fundus examination showed macular lesions in all patients. We found yellowish macular deposits in 2 patients (F6 and F7), macular atrophy in 3 patients (F8, F11), beaten-bronze macula in 3 patients (F9) and symmetrical cloverleaf maculopathy in three patients (F10). Peripheral retina showed nummular pigment deposits in 2 patients (F6 and F7), few bone spicule shaped deposits in the mid periphery along with atrophy of the periphery in 8 patients (F8, F9 and F10), and hyperplasia of the retinal pigment epithelium (RPE) with yellowish deposits and atrophy in 5 patients (F11) (Fig. 3).

##### F8-*CDHR1* (Fig. 3C, D, E, F and G)

A novel homozygous deletion (NM_033100: c.863-2_863-1delAG, p.?) in *CDHR1* was observed in F8 with no other candidate P/LP variants (Fig. 2). This deletion is located in the crucial splice acceptor domain of intron 9 and could impact the normal splicing pattern of *CDHR1.*

##### F9-*C8ORF37* (Fig. 3H, I, J, K, L, M, N, O and P)

The affected individual in family F9, carried a novel homozygous splice-site pathogenic variant (NM_177965: c.470 + 1G > T, p.?) in *C8ORF37* (Fig. 2). This gene has recently been shown to cause RP^7^, with only 5 cases reported with splice-site variants. This variant was located in the donor splice site of intron 6.

##### F11-ABCA4

**Figure 4:**
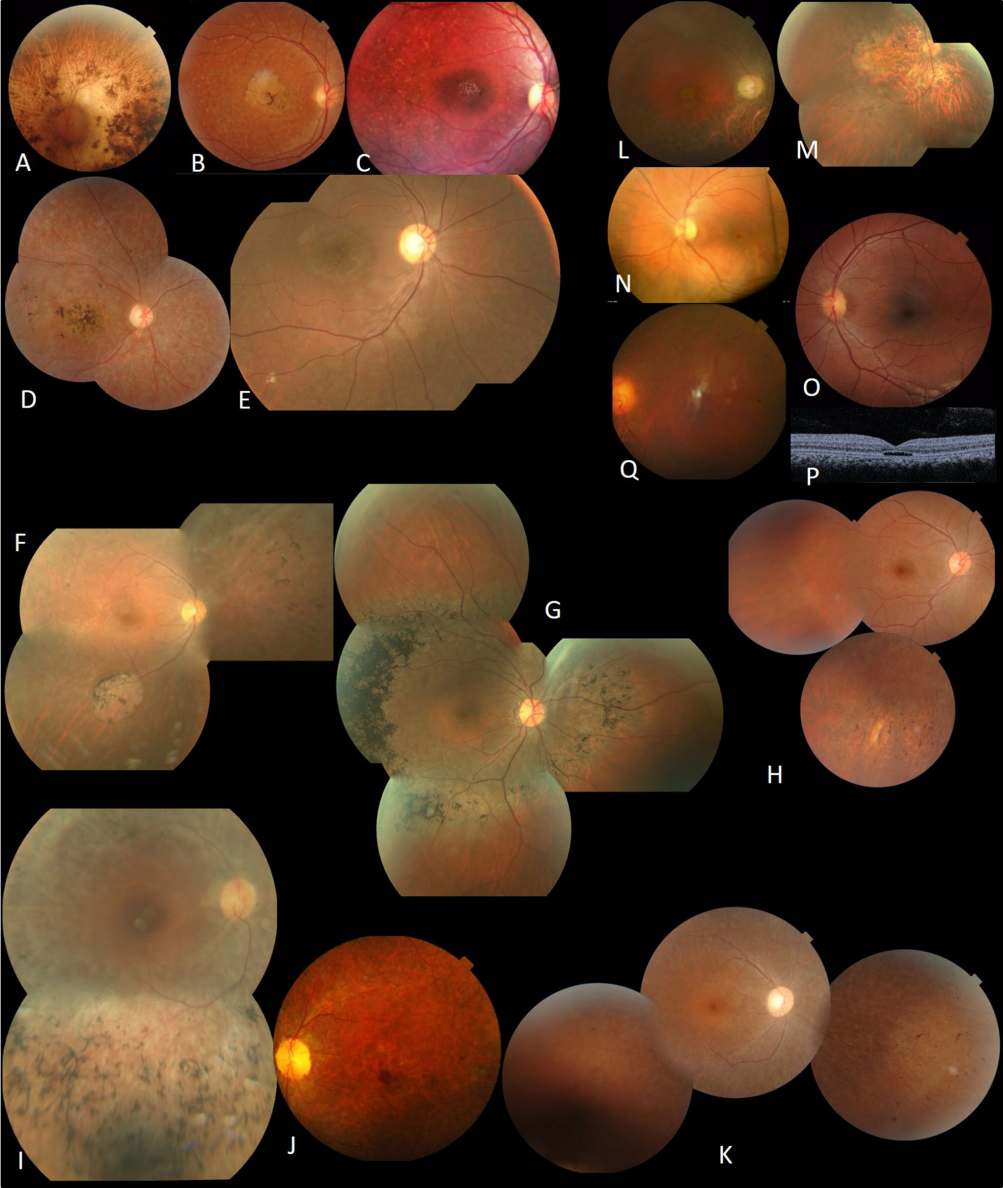
Clinical features of patients with *ABCA4* pathogenic variant (**A**, **B**, **C**, **D** and **E**); RP patients (**F**, **G**, **H**, **I**, **J** and **K**); BB patients (L,M); achromatopsia (**N**, **O**, **P**) and CSNB (**Q**). **A** F11, LE fundus of patient II.2. **B** F12, RE fundus of patient II.1. **C** F13, RE fundus of patient II.2. **D** F14, RE fundus of patient III.3. **E** F15, RE fundus of patient II.1. **F** F16, RE fundus of patient V.4. **G** F17, RE fundus of patient V.1. **H** F18, RE fundus of patient IV.1. **I** F19, RE fundus of patient IV.2. **J** F20, LE fundus of patient II.5. **K** F21, RE fundus of patient II.5. **L** F22, RE fundus of patient II.3. **M** F23; RE fundus of patient II.1. **N** F24, LE fundus of patient II.3. **O** F25, LE fundus of patient II.3. **P** F25, LE OCT of patient II.3. **Q** F26, LE fundus of patient II.1.

A novel homozygous likely pathogenic variant (NM_000350.2: c.1916A > G, p.Y639C) in exon 13 in *ABCA4* was identified in family F11 (Fig.4). Clinical data showed typical hallmarks of CRD.

#### RP Patients (Fig. 2,4)

The 10 patients (F16, F17, F18, F19, F20 and F21) with RP had a mean age of 35.57 years (21–63 years) with disease onset ranging from 5 to 20 years. All patients had night-blindness. BCVA ranged from hand movement to 10/10. Fundus examination showed typical RP with bone spicule deposits in mid periphery in all patients. In 2 patients, we found large areas of atrophy (F16). Macula was normal in 6 patients and atrophic in four (Fig. 4F).

##### F16-*NMNAT1* (Fig. 4F).

The homozygous pathogenic variant (NM_001297778.1: c.37G > A, p.A13T) in *NMNAT1* segregated with the disease in F16, with two affected members (F16-V.1, F16-V.4) (Fig. 2). This pathogenic variant has previously been reported as causing LCA^8^. In contrary to this report, our affected patients had relatively preserved visual acuity until the third decade (Table 2). On fundus examination, we found normal macular aspect with few bone spicule shaped pigment deposits and white dot deposits, large areas of atrophy in the mid periphery (Fig. 4F).

##### Bardet-Biedl (Fig. 2,4)

Clinical reassessment of extraocular symptoms was also performed in 4 patients from two families (F22, F23) who were shown to have Bardet-Biedl syndrome with retinal dystrophy, obesity and polydactyly. Fundus examination showed macular atrophy in all patients with bone spicule deposits and atrophy in the peripheral retina (Fig. 4M and N).

##### Other retinal dystrophies (Fig. 2, 4)

Four patients (F24, F25) had achromatopsia with nystagmus, photophobia and visual impairment since birth. Fundus examination was normal and OCT showed optically empty space with partial retinal pigment epithelium disruption in 2 patients (F25) (Fig. 4O and P). The index patients (F26) had a story of nyctalopia since childhood. Ophthalmic examination showed high myopia, cataract and chorioretinal atrophy (Fig. 4R). A novel likely pathogenic variant NM_002420.5: c.3947 T > G, p.L1316R in *TRPM1* (Fig. 2) was identified in the index patient in F26.

## Discussion

**Figure 5:**
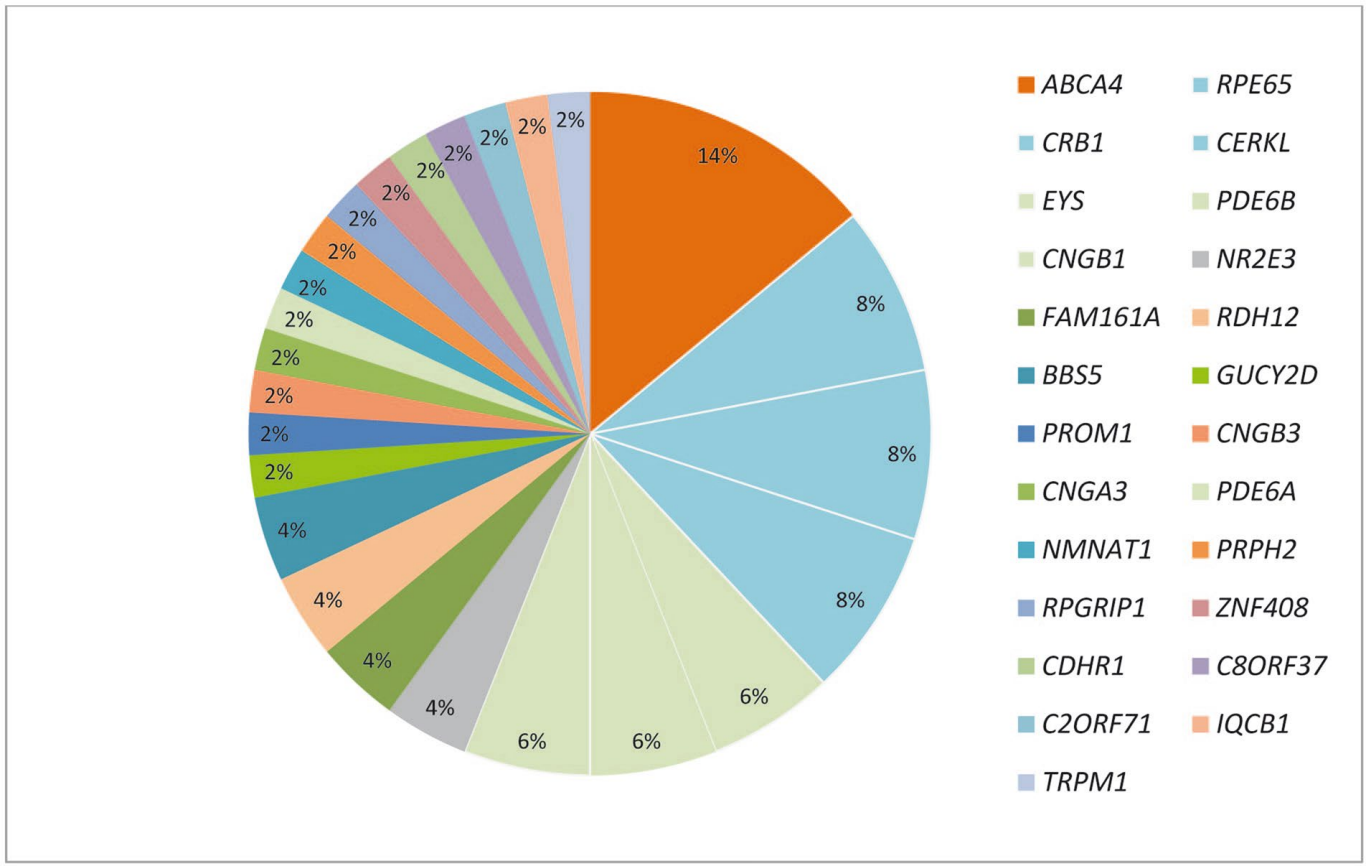
Mutational spectrum in 73 Tunisian cases with confirmed molecular diagnosis.

The data presented here showed that a number of genes can cause IRD in this Tunisian cohort. Taking together with our previous report, the analysis of 73 Tunisian families highlights the mutational load in IRD by identifying likely disease-causing genes in more than 25 genes in 50 families associated with different forms of IRD. A total of 50 likely disease causing alleles were identified, including 8 nonsense pathogenic variants, 10 deletions, 1 duplication, 1 complex rearrangement, 6 splice-site alleles and 24 missense alleles considered potentially pathogenic, 42% of which were novel. In addition, a novel finding from this study was the evidence of high frequency of *ABCA4*, *RPE65*, *CRB1* and *CERKL* pathogenic variants in Tunisian families with IRD^9–12^ (Fig. 5). Homozygosity mapping combined with systematic screening of known genes resulted in a positive molecular diagnosis in 68.4% families. This is in accordance with several reports^13,14^ and is similar to the 75% frequency reported in Saudi Arabia^15^. However, in Spanish cohort studying large sporadic IRD groups (877 patients), the diagnostic yield was 44%^16^. On one hand, this difference might be explained by the selection of the cohort analyzed, as we chose families with either two affected individuals or sporadic cases with particular phenotype and we excluded patients with Usher syndrome. On the other hand, this may be due to the high frequency of consanguineous marriages in our cohort.

This work provides an overview of the mutational spectrum of IRD in Tunisian cohort (Fig. 5) which gives an the most frequent genes in our cohort of patients with retinal disorders were: 14% *ABCA4* (p.E1087K, p.W782*, p.Y639C, p.P1380L, p.N380K and dup32-40; del45-47), 8% *RPE65* (p.R91W, p.H182Y, p.R234* and c.1129-2A > G), 8% *CRB1* (p.R764H, p.P836T, p.Y702C and c.3542 + 1G > A) with variable phenotypes of severe IRD, ranging from LCA to RP as previously reported^17^ and 8% *CERKL* (c.1133 + 3_1133 + 6delAAGT). As expected, our results look more similar to the Spanish cohort where *CRB1* (7%), *ABCA4* (7%), *CERKL* (4%) and *EYS* (4%) were the most frequent mutated genes^16^. Compared to other ethnic groups, however, the most prevalent pathogenic genes in Saudi Arabia were *KCNV2, RP1, TULP1, RPGRIP1, CRB1* and *RPE65* respectively^15^. In addition, characterized patients in Israeli/ Palestinian populations show high frequency of pathogenic variants in *FAM161A*, *CRB1*, *USH1C*, *MAK* and *DHDDS*^17^. This could be explained by the two countries sharing some ethnic origins.

In this study, we also highlighted the importance of combining molecular and clinical data to correctly diagnose IRDs. We would like to point out that in the subset of families analyzed in this study potential disease-causing variants were detected in 19 genes, out of which 8 have not yet been described in association with the observed IRD phenotype: *RPGRIP1, GUCY2D*, *CRB1*, *CDHR1*, *C8ORF37*, *ABCA4*, *CNGB1* and *TRPM1*. Ophthalmic and genetic findings are presented in Table 3.

**Table 3:** Description of the new pathogenic variants identified in our cohort.

Phenotype	F	Gene	New pathogenic variant	Phenotypes	Literatures	Hypothesis /note
LCA	F1	*RPGRIP1*	c.3113_3114delCT	visual acuity was limited to light perception	Several studies have shown that patients with *RPGRIP1* pathogenic variants have a greater variation in phenotype severity depending on the localization of the variants^44^	Most LCA-associated pathogenic variants are located in a segment that encodes two C2 domains^45^. Some RP- and LCA-causing pathogenic variants in either RHD or RID were shown to impair the interaction between the two^46^. These data may explain this phenotypic variation in our patient
	F2	*GUCY2D*	c.2660 T > G	severe visual dysfunctions	70% of families with LCA caused by pathogenic variants in *GUCY2D* originate from Mediterranean countries^47^	This protein is involved in ciliary transport and abnormal trafficking was associated with the most severe visual dysfunctions (LP, NLP at birth)^48^ which are similar to those described in members of family F2
	F3	*CRB1*	c.3542 + 1G > A	LCA	*CRB1*-linked pathogenic variants cause specific fundus features: preservation of the para-arteriolar retinal pigment epithelium and retinal telangiectasia with exudation^48^ but this may not be exclusive	The presence of novel *CRB1* pathogenic variants in our cohort expands the mutation spectrum of *CRB1*
CRD	F8	*CDHR1*	c.863-2_863-1delAG	CRD	Previous reports showed that the majority of *CDHR1* pathogenic variants likely result in nonsense mediated mRNA decay^49^	A recent study demonstrated that pathogenic variants in *CDHR1* lead both to RP and CD or CRD^49^. Stingl et al. proposed that an early maculopathy might be a symptom to be expected in all patients with CDHR1-related retinopathy regardless of age^49^, as found in our patients
	F9	*C8ORF37*	c.470 + 1G > T	Constant early macular involvement and a variable phenotype depending on the age	Pathogenic variants in *C8ORF37* is a rare cause of IRD (0,4% in Pakistani cohort)^50^	The phenotype of the patients shows broad variability ranging from CRD to RP with early macular involvement^51,52^ to syndromic conditions: Bardet-Biedl syndrome (BBS)^53^
	F11	*ABCA4*	c.1916A > G	CRD	According to several studies there is a frequent ‘ethnic group-specific’ *ABCA4* alleles^54,55^, however, populations outside of Europe are comparatively less well-characterized	The most frequent variant observed in our Tunisian cohort is p.E1087K. Our result needs to be confirmed by analyzing more cases with STGD
RP	F19	*CNGB1*	c.2293C > T	AR-RP	There is a gene-phenotype relationship between *CNGB1* and ar-RP^56^ which is consistent with our results	This expands the spectrum of *CNGB1* variants in RP cases
CSNB	F26	*TRPM1*	c.3947 T > G	CSNB	More than 35 pathogenic variants in *TRPM1* are found in approximately half of all patients with complete congenital stationary night blindness (cCSNB)^57^	High myopia has been consistently reported, similarly to the clinical data of our index patient^58^

LCA = Leber congenital amaurosis; CRD = cone rod dystrophy; RP = retinitis pigmentosa; CSNB = congenital stationary night blindness; AR-RP = autosomal recessive retinitis pigmentosa; IRD = Inherited retinal dystrophies; STGD = Stargardt disease; LP = Light perception; NLP = No light perception.

Ophthalmic investigation identified characteristic signs and symptoms of LCA in five families (F1, F2, F3, F4 and F5). This group of the most severe and the earliest occurring IRD resulting in congenital blindness^18^ typically becomes evident in the first year of life like in our five families represent 19.2% of our cohort. To date, genetic heterogeneity of LCA is well known, with 24 genes currently implicated in its pathogenesis^19^. Molecular analysis in our families identified three new pathogenic variants: novel homozygous deletion c.3113_3114delCT identified in *RPGRIP1*. The second missense pathogenic variant p.V887G is localized in *GUCY2D*, gene, estimated to account for 20% of LCA cases^20^ and constitute the most common cause of the disease. The third is a novel splice variant c.3542 + 1G > A in *CRB1*, the most commonly mutated gene in our cohort (8%) with variable phenotypes of severe IRD, ranging from LCA to RP as previously reported^21^.

NGS allows for the screening of a large number of genes implicated in the pathophysiology of IRD. In F4, despite having a previously reported homozygous pathogenic variant p.R332* in *IQCB1*, the phenotypes of our index patient and their affected sister never showed dysplasia in any organ; rather, they only had LCA. Usually, defects in this gene result in Senior-Loken syndrome type 5 (SLSN5), where degenerative phenotypes involving kidneys and eyes are common^22^, but sometimes the phenotype only shows LCA, as presented in this family^23,24^.

We report ophthalmic and genetic findings of patients with RP, composed of 10 patients, with AR-RP presented in 8 patients and 2 patients with AD-RP phenotype, which represent 23% of our cohort. patients with AR-RP were carrying four new pathogenic variants: Patients in F8 were carrying a novel homozygous deletion c.863-2_863-1delAG localized in the crucial splice acceptor domain of intron 9 *CDHR1*. To date, studies have revealed around thirty cases with this particular *CDHR1* pathogenic variant; gene known to play a key role in the maintenance of photoreceptor structure and integrity^25^. We also describe a novel homozygous splicing pathogenic variant c.470 + 1G > T in *C8orf37*, observed in F9. The localization of genetic abnormalities has previously been described by Rahner et al., where 56% of the pathogenic variants are located in exon 6 in the C-terminal region of *C8orf37* and the majority of reported variants are splicing variants^26^. We identified new homozygous pathogenic variant p.Y639C in family F11. *ABCA4* pathogenic variants were responsible for 14% of cases in our cohort for a wide variety of IRD phenotypes from AR Stargardt disease to CRD and, in some advanced cases RP^27^. Therefore, no clear genotype–phenotype could be established. Three different pathogenic variants were identified in *CNGB1*, which represent 6% of our cohort. One of these variants is a novel homozygous pathogenic variant p.R765C in F19 where the index patient presented typical symptoms of RP.

Unexpectedly, two probands in family F16 with RP had damaging missense pathogenic variant, p.A13T in nicotinamide adenine dinucleotide (NAD) synthase gene *NMNAT1*. This pathogenic variant has been previously identified in patients with LCA^8^. Although the proband’s phenotype is consistent with RP, and the pathogenic variants are predicted to be deleterious, our patients showed well preserved visual acuity. Fundus examination revealed bone spicule-like pigment deposits and white spot deposits at the mid-periphery.

Our data provide an overview of the mutational spectrum of IRD in a Tunisian cohort, which gives an idea about genes spectrum in North Africa. We demonstrate a high degree of genetic complexity in both, the causative disease genes and their associated phenotypes, highlighting uncommon genotype–phenotype correlations and contributing to the current knowledge about disease-causing variants. We realise that this study presents some limitations, such as relatively small number of patients and the lack of complete ophthalmic and other examinations. Ideally, the efficacy of genotype–phenotype correlation could be improved with a complete ophthalmic examination, including ERG in all patients.

## Methods

### Clinical data and sample collection

150 families were evaluated at the Department B of Hedi Rais Institute of Ophthalmology, Tunis; Tunisia, over the course of 15 years. We selected a subset of families which accepted to be part of the study, with onset of the disease in the first or second decade of life, with clinical diagnosis of AR-IRD excluding patients with unclear or unlabeled diagnosis of retinal dystrophy or for whom multimodal imaging exploration could not be carried out. 73 families fulfilled these criteria.

In this study, we draw up a report on 26 families. Consanguinity involving first-cousin marriages was observed, and in the non-consanguineous families, most marriages were between individuals from the same geographic origin and the highest number of cases was recorded in the region of Nabeul containing 15 families. A questionnaire was used to collect information which included socio-demographic data (age, gender, geographical origin, educational level, occupation, socio-economic level) family medical, surgical and ophthalmological history, age of onset and duration of symptomatology (onset date has been defined by the age of onset of the first visual symptoms), the disease course (defined as either stationary or progressive) and any additional non-ocular findings, such as deafness, mental retardation, polydactyly, obesity, heart disease or other malformations. Each patient had complete ophthalmological examination including best corrected visual acuity (BCVA) using Snellen chart, slit-lamp biomicroscopy, dilated fundus examination, retino-photos, optical coherence tomography (Swept source DRI OCT-A Triton®,Topcon, Tokyo, Japan), fundus autofluorescence (Heidelberg Spectralis; Heidelberg-Engineering, Heidelberg, Germany) and some patients also received full-field electroretinogram (ERG) (Métrovision, France).

This study was approved by the Local Ethics Committee of the Hedi Rais Institute. Peripheral blood samples were obtained from the index patient and from some of the family members, including parents and affected siblings. Informed consent was obtained from all participants and a parent and/or legal guardian for participants under the age of 18 years old. Analyses were done in accordance with local guidelines. DNA was extracted from leukocytes according to the salting-out method^28^.

### Whole exome sequencing (WES)

Exome capture was performed using the Roche Nimble-Gen version 2 (44.1–megabase pair) and paired-end multiplexed sequencing was carried out on an Illumina HiSeq 2000 system (Illumina, San Diego, CA, USA) by Otogenetics Corporation (Norcross, Georgia, USA) using DNA samples from all index cases. Homozygosity was evaluated from SNPs obtained by WES.

### Sequence data alignment, variant calling and identification

The Illumina paired-end DNA sequence data were mapped and aligned to the reference human genome NCBI Build 37 (hg19) using the Nextgene software package v.2.3.5. (Soft-genetics, State College, PA). Median coverage of the target region was 95X with 96% of target region covered by at least 10 reads.

### Variant assessment

Identified variants were analyzed by PolyPhen-2 (https://genetics.bwh.harvard.edu/pph2/) and SIFT (https://sift.bii.a-star.edu.sg/) to predict the pathogenicity of the respective variants. The variant frequency in the healthy control population was evaluated using ExAC (https://exac.broadinstitute.org/) and gnomAD (https://gnomad.broadinstitute.org/) databases. Variants outside exons and flanking splice regions, synonymous or with a minor allele frequency (MAF) > 1% were filtered out. Amino acid conservation of the altered protein region was analyzed using a multispecies alignment comparing human, monkeys, chicken, fish, frog, fly and worm. Protein sequences were obtained from Uniprot (https://www.uniprot.org) or PolyPhen-2.

To predict the putative impact of the identified splice site variation, in silico analysis was done using Human Splicing Finder (v2.4.1), SKIPPY (https://research.nhgri.nih.gov/skippy) and the Automated Splice Site Analyses (https://www.fruitfly.org/seq_tools/splice.html).

The resulting list of homozygous gene variants was compared to the IRD genes found in RetNet (https://sph.uth.edu/retnet/disease.htm).

### Sanger sequencing

Identified variants were validated by Sanger sequencing and segregation analysis. DNA was amplified by PCR using FastStart PCR Master Kit (Roche, Basel, S) and sequenced as previously described^11^. Primers and condition of each PCR are provided in Table 4. Fragments were sequenced on an ABI 3100XL DNA automated sequencer (Applied Biosystems, Foster City, CA).

**Table 4:** Sequences of primers and PCR condition.

*Genes*	Exon	Forward	Reverse	Hybridation temp. (°C)
*RPGRIP1*	19	AAAGAAGGCAGGAAGGAAGG	TCTTGAAAGCCTGATCTCGTG	58 (bet)
*GUCY2D*	14	GACCGGCTGCTTACACAGAT	GACAGGAGGTCTGGGAAAGA	58
*RPE65*	7	GCCTGTATAAGCTGTTCTAA	CTCAGTTACAAGAATCAACAG	60
*IQCB1*	11	AGATTGCACAACAGCAGCAG	CAGAGAAAAAGGACAAAAGTCCA	60
*CRYBA1*	5	TTTCTCACAAATCTGTTGCCTTA	CCGATACGT ATGAAATC TGATTAAAA	58
*CRB1*	10	CCTCCAGCAGGAGCTTTTTA	GCATAGATTTTCCTATGGGAACTG	60
*CRB1*	7bb	GCTGACTCCAAACTCTCCCA	TGGTGGGTCAGTAACATGATCT	60
*CRB1*	6b	GCAACAGGGATGTGTTTGTG	TTTCATAGCAGGCAGAAGCA	65
*CDHR1*	10	GGGAGCTGGACAGAAGTGAG	CACCTCCTCTTGCCTTTCTG	58
*C8ORF37*	5	CAGTAATCTGTAATATGTGGTGTATCC	CCCACAAGATCTGGCTGAAT	58
*C2ORF71*	1E	GCAGCAAGTCCACAGAGAAC	TGTAAGAGGAGGGAAGGCTC	58
*ABCA4*	13	GGTGAGAGTCTGATACCTCT	AGCCAACTCGAAATGGCTCT	58 (bet)
*ABCA4*	28	GGCTTGACTACTTCCATAGC	AGGTTACATGGACCTCAGCT	58 (bet)
*ABCA4*	9	TCCATGGAAGCAGTGACTTTT	CAATGTCACTCATTATCTTCAGCA	58
*ABCA4*	22	ATACGTGACCATGGAGCTTG	AACAAGCTCATCTGACCAGG	58
*NMNAT1*	2	TGGCAGAGCAAGACCTTATC	GGACTACAGGCACAGTGAAT	60
*PRPH2*	1	TCGTTAAGGTTTGGGGTGGGA	CTGGTCAGAGGCCTGAGCCT	58
*FAM161A*	3	TGGTCACATACAACTGAAAGTATAAATAACA	GCTTCTGTTCCTCCCTTGCT	60
*CNGB1*	23	AGAGACTCCGCCTCTCACTC	GGGGCAGACACGAAGATG	58
*EYS*	29	AATCTGCTTCTGGCTTTGTTT	GCCCCACTAGCCAGAAAATA	58
*EYS*	12	TTTTTAAATGCACCCCACAA	ACCAATCAATAGACACATTTGAGA	58
*BBS5*	4	AGGAGACAGAATTGACCCTCT	CATGAAACTGGTCCCTGGTG	58
*BBS5*	2	AAATGCATGAACATTTGGTACA	CACAATTACACTGACAAATGATGC	58
*CNGA3*	8A	GCTGTGGCAGCATTACAAGA	GAATCAATCTTGGCCTGGAA	58
*CNGB3*	16	CACCTGGACCCTCACCTCTA	CGGTTCTCCCTATCTCAGAGT	58
*TRPM1*	27A	TTCTGAAAAATCACATAGCAATGAC	CGTTTCCACTGTTAGCTGAGTG	58
